# Supplementary material for: Deep Learning Methods Applied to Drug Concentration Prediction of Olanzapine
Source: Pharmaceutics. 2023 Apr 4;15(4):1139. doi: 10.3390/pharmaceutics15041139 (PMC10145228; doi:10.3390/pharmaceutics15041139)
Supplement: Supplementary file 1 [file pharmaceutics-15-01139-s001.zip › pharmaceutics-2278213-supplementary.pdf]

Supplemental Table S1. Parameters of the Bayesian Optimization Algorithms

Package	Surrogate Model	Acquisition Function	Number of Trials	Direction	Loss Function
Sci-kit Optimize	Gaussian Process<br>Random Forest<br>Gradient Boosted Trees<br>Random Search	Expected Improvement	20	Minimize	Mean Squared Error
Optuna	Tree Structured Parzen Estimators with Hyperband Pruner	Expected Improvement	20	Minimize	Mean Squared Error

Supplemental Table S2. Readings from each of the hyperparameter optimization algorithms

Models	Number of L and A layers	Training MSE	Testing MSE	Training RMSE	Testing RMSE
Tree Structured Parzen Estimators with Hyperband Pruner	LSTM1ANN1	367.899	912.905	19.181	30.214
	LSTM1ANN2	343.481	873.53	18.533	29.556
	LSTM1ANN3	455.955	931.309	21.353	30.517
	LSTM2ANN1	399.748	919.197	19.994	30.302
	LSTM2ANN2	354.938	951.785	18.840	30.851
	LSTM2ANN3	599.12	841.295	24.477	29.005
	LSTM3ANN1	480.403	879.11	21.918	29.650
	LSTM3ANN2	443.173	910.915	21.052	30.181
	LSTM3ANN3	405.67	920.319	20.141	30.337
Gaussian Process	LSTM1ANN1	534.001	907.881	23.108	30.131
	LSTM1ANN2	437.399	950.627	20.914	30.832
	LSTM1ANN3	271.937	986.318	16.491	31.406
	LSTM2ANN1	423.955	891.941	20.590	29.865
	LSTM2ANN2	404.292	870.469	20.107	29.504
	LSTM2ANN3	420.726	908.186	20.512	30.136
	LSTM3ANN1	425.911	882.381	20.638	29.705
	LSTM3ANN2	388.991	901.757	19.723	30.029
	LSTM3ANN3	408.874	876.527	20.221	29.606
Random Forest	LSTM1ANN1	423.527	922.805	20.573	30.378
	LSTM1ANN2	258.520	1027.620	16.079	32.057
	LSTM1ANN3	272.105	951.971	16.496	30.540
	LSTM2ANN1	365.503	957.418	19.118	30.942
	LSTM2ANN2	360.218	953.608	18.979	30.881
	LSTM2ANN3	461.560	926.311	21.484	30.435
	LSTM3ANN1	374.541	879.306	19.353	29.955
	LSTM3ANN2	335.554	866.907	18.236	29.443
	LSTM3ANN3	471.646	902.648	21.717	30.044

Gradient Boosted Trees	LSTM1ANN1	245.262	1014.778	15.661	31.856
	LSTM1ANN2	393.817	941.132	19.845	30.678
	LSTM1ANN3	343.515	917.030	18.534	30.283
	LSTM2ANN1	437.909	949.287	20.926	30.811
	LSTM2ANN2	331.613	967.444	18.210	31.104
	LSTM2ANN3	433.662	905.407	20.825	30.090
	LSTM3ANN1	377.004	899.096	19.417	29.985
	LSTM3ANN2	383.052	896.525	19.572	29.942
	LSTM3ANN3	203.945	959.406	14.281	30.974
Random Search	LSTM1ANN1	443.572	938.193	21.061	30.630
	LSTM1ANN2	462.634	967.990	21.509	31.113
	LSTM1ANN3	323.297	1028.026	17.980	32.063
	LSTM2ANN1	501.100	907.351	22.385	30.122
	LSTM2ANN2	381.616	952.065	19.535	30.856
	LSTM2ANN3	433.662	905.407	20.825	30.090
	LSTM3ANN1	377.004	899.096	19.417	29.985
	LSTM3ANN2	503.652	895.531	22.442	29.925
	LSTM3ANN3	471.713	919.307	21.719	30.320

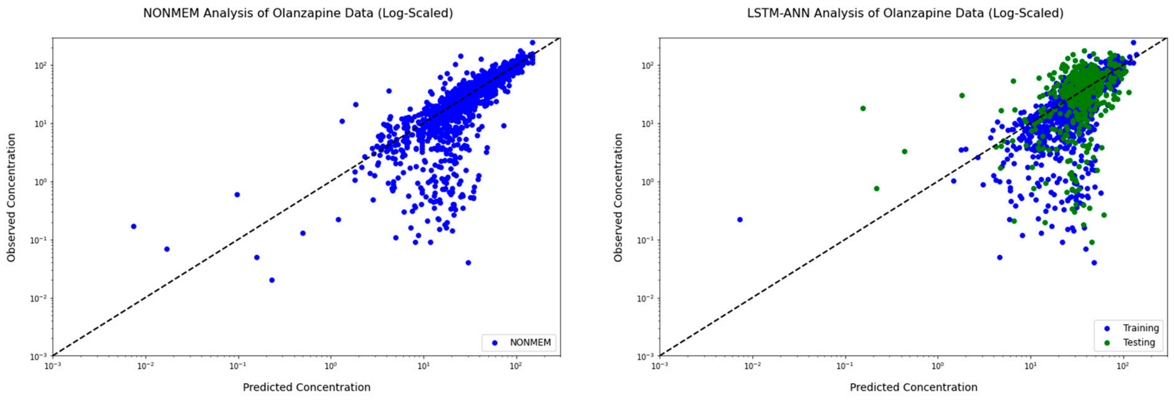

(**a**) (**b**)

Supplemental Figure S1. Observations vs. Prediction plot in a log scale from the different modeling approaches. (**a**) NONMEM (note: predictions are individual predictions [IPRED]); (**b**) LSTM-ANN (The different colors correspond to drug concentration predictions from the different datasets. Blue represents the training data, and the green represents the validation data.)
